# Clinic-based prevalence of uncorrected refractive errors and associated visual impairment: a nationwide cross-sectional study of 3.6 million outpatients in Mexico

**DOI:** 10.1186/s12889-026-26598-9

**Published:** 2026-02-20

**Authors:** Abraham García-Gil, Beatriz Itzel Hernández-Jurado, Marco Antonio Luna-Ruiz-Esparza, Perla Lorena Ayón-Sicaeros, Eduardo Espinoza-Angulo, Héctor Machado-Jiménez, Humberto Gómez-Campaña, Linda Nasser-Nasser, Abraham Campos-Romero, Jonathan Alcántar-Fernández

**Affiliations:** 1 Innovation and Research Department, Salud Digna, Culiacan, 80184 Sinaloa Mexico; 2 Optometry Department, Salud Digna, Culiacan, 80184 Sinaloa Mexico; 3 Medical Direction, Salud Digna, Culiacan, 80184 Mexico; 4 Medical Direction, Visión Cirugía Ambulatoria S.C, Monterrey, 64710 Nuevo Leon Mexico

**Keywords:** Uncorrected refractive errors, Myopia, Astigmatism, Hyperopia.

## Abstract

**Background:**

Uncorrected refractive errors (UREs) are the predominant cause of vision impairment and blindness worldwide, despite the availability of eyeglasses as a cost-effective intervention. Sociocultural factors, including restricted access to healthcare and inadequate self-care education, exacerbate the burden of UREs. There is a paucity of studies evaluating UREs in Mexico with comprehensive national coverage across various age groups. This clinic-based study aimed to assess the profile of UREs and associated visual impairment (VI) in Mexico using a substantial sample of individuals seeking care at a large nationwide outpatient network.

**Methods:**

We evaluated UREs and URE-related VI by analyzing eye examination data from 3.6 million outpatients who attended Salud Digna clinics in 2024.

**Results:**

The national clinic-based prevalence of UREs was 26.5% (980,718 affected individuals, 95% CI:26.5%-26.59%), highest in Baja California Sur state (6,515 affected individuals, 43.8%, 95% CI:42.99%-44.59%), among males aged 40–49 years (71,002 affected individuals, 34.1%, 95% CI:33.91%-34.32%), and females under 10 years (17,132 affected individuals, 32.3%, 95% CI:31.88%-32.67%). Clinic-based prevalence of uncorrected astigmatism, uncorrected hyperopia, uncorrected hyperopia-astigmatism, uncorrected myopia, and uncorrected myopia-astigmatism were 1.3% (48,978 affected individuals, 95% CI:1.31%-1.34%), 10.2% (376,701 affected individuals, 95% CI:10.16%-10.23%), 1.7% (61,171 affected individuals, 95% CI:1.64%-1.67%), 6.4% (235,285 affected individuals, 95% CI:6.34%-6.39%), and 7% (258,583 affected individuals, 95% CI:6.97%-7.02%), with highest rates in Tlaxcala (444 affected individuals, 3.9%, 95% CI:3.54%-4.26%), Tabasco (10,235 affected individuals, 20%, 95% CI:19.66%-20.35%), Baja California Sur (673 affected individuals, 4.5%, 95% CI:4.2%-4.87%), Zacatecas (3,149 affected individuals, 10.2%, 95% CI:9.85%-10.52%), and Tlaxcala (1,504 affected individuals, 13.17%, 95% CI:12.55%-13.8%).

The national URE-related VI clinic-based prevalence was 0.89% (32,962 affected individuals, 95% CI:0.88%-0.9%), highest in Hidalgo state (527 affected individuals, 1.8%, 95% CI:1.63%-1.93%) and among those over 80 years (1,457 affected males, 6%, 95% CI:5.97%-6.59%; 2,206 affected females, 5.8%, 5.83%-6.33%). The national clinic-based prevalence of mild, moderate, and severe visual impairment was 0.62% (22,858 affected individuals, 95% CI:0.61%-0.63%), 0.26% (9,683, 95% CI:0.26%-0.27%), and 0.01% (421 affected individuals, 95% CI:0.01%-0.01%).

**Conclusions:**

This outpatient-based study highlights the clinic-based prevalence of UREs and related VI in Mexico, emphasizing the need for improved access to eye care services.

**Supplementary Information:**

The online version contains supplementary material available at 10.1186/s12889-026-26598-9.

## Background

Globally, more than one billion individuals live with vision impairment that is either preventable or unaddressed. In 2020, an estimated 295 million people were affected by moderate to severe visual impairment (VI), with projections indicating an increase to 474 million by 2030. Uncorrected refractive errors (UREs) are the leading cause of this burden, disproportionately affecting older adults in low- and middle-income countries [1–3]. Refractive errors result from a mismatch between the axial length of the eye and its optical power, producing blurred retinal images. The three primary types of refractive errors include hyperopia (farsightedness) and myopia (nearsightedness), which are classified as spherical errors [4, 5], and astigmatism, which arises from optical asymmetry. Although astigmatism is typically considered a distinct category, it frequently coexists with either hyperopia or myopia [6].

Although refractive errors are not curable, timely detection and appropriate clinical intervention can effectively mitigate vision impairment through corrective measUREs such as eyeglasses, contact lenses, or refractive surgery [7, 8]. Nearly all human eyes exhibit some degree of refractive error, regardless of whether optical correction is used [6]. However, it is estimated that globally, only 36% of individuals with refractive errors have access to appropriate eyeglasses, despite their proven cost-effectiveness as a public health intervention [9].

Beyond the direct impact on vision, UREs carry substantial social and economic consequences, limiting educational attainment, employment opportunities, and overall productivity [10]. In 2007, the global productivity loss attributable to VI from uncorrected or undercorrected refractive errors was estimated at 121.4 billion international dollars, excluding individuals aged over 50 years [11].

While global data underscore the magnitude of visual impairment, national statistics reveal a substantial burden in Mexico. According to the 2020 Population and Housing Census, 2,691,338 individuals reported having a VI [12]. Additional estimates suggest that approximately 11.01 million Mexicans (8.7% of the population) are either blind or visually impaired, with refractive errors accounting for 2.61 million of these cases [13]. Despite this burden, nationally representative studies encompassing all age groups remain scarce. Among the few available, Terán et al. [14, 15] conducted local assessments of the three major refractive errors. In Northwestern Mexican students aged 12–15 years, the prevalence of uncorrected myopia (UM), uncorrected astigmatism (UA) 19.8%, and uncorrected hyperopia (UH) was 24.7%, 19.8%, and 2.0%, respectively. Among students aged 15–18 years, the rates increased to 36.1%, 29.2%, and 1.5% for UM, UA, and UH, respectively.

Since 2015, Salud Digna, a non-profit organization operating over 200 outpatient facilities nationwide, has been the leading provider of eyeglasses in Mexico. This study utilized eye examination data from Salud Digna to primarily estimate the clinic-based prevalence and geographic distribution of UREs. Additionally, the secondary objectives included examining disparities based on sex and age, as well as assessing the burden associated with VI. These findings are intended to inform national strategies for improving access to visual health services and guide policy development in alignment with WHO recommendations [10].

## Methods and materials

### Study population

In this cross-sectional retrospective study, we analyzed anonymized electronic health records from non-cycloplegic eye examinations conducted at Salud Digna outpatient clinics across all 32 Mexican states between January 1 and December 31, 2024. All examinations were performed by certified optometrists following a standardized protocol. Initially, the patient’s identity was verified, and a clinical history form was completed. All instruments were sterilized before use. Measurements of interpupillary and nasopupillary distances were conducted, and uncorrected visual acuity was assessed using digital optotypes positioned at a distance of 3 m (CS Pola 600, CS 550, Essilor Instruments, Paris, France). An autorefractor (AKR550 and WAM 800; Essilor Instruments, Paris, France) was used to obtain objective refraction, which was subsequently refined to determine the prescription and evaluate both distance and near visual acuity. Examination findings and recommendations were communicated, and referrals for ophthalmology or retinal imaging were provided as necessary.

Demographic and clinical data were collected using a validated uniform survey instrument implemented across all facilities.

Individuals with documented ocular comorbidities that could independently cause visual impairment, such as macular disorders, glaucoma, or cataracts, were excluded from the analysis. This exclusion was based on diagnostic codes and clinical notes recorded in the electronic health records.

For the analysis of UREs clinic-based prevalence, data from the most affected eye were used; in cases where both eyes were equally affected, the right eye was selected. Visual impairment (VI) was evaluated using the best-corrected visual acuity (BCVA) in accordance with the World Health Organization (WHO) guidelines [10] solely for descriptive purposes; no uncorrected visual acuity (UCVA) data were used.

Refractive errors were defined according to Galvis et al. [5] as follows: myopia, ≤ −0.50 diopters (D) spherical equivalent (SE); hyperopia, ≥ +0.50 D SE; and astigmatism, ≤ −1.00 D cylinder. URE was defined as a refractive error that could be corrected with eyeglasses but remained unaddressed [16].

### Statistical analysis

Descriptive statistical analyses were conducted using Microsoft Power Query and Microsoft Excel to summarize the demographic and clinical characteristics of the study population. All inferential statistical analyses were performed using R (version 4.4.0) [17]. The primary analyses focused on estimating the overall clinic-based prevalence of UREs and VI. These estimates were calculated using the dplyr package (v1.1.4) and the epitools package (v0.5-10.1). Exact 95% confidence intervals (CIs) for prevalence proportions were computed using the binom.exact() function from the epitools package, which applies the Clopper-Pearson method based on binomial distribution.

Exploratory analyses included stratification by sex and age, as well as a breakdown by the type of refractive error and severity. Chi-square tests for independence were conducted using the chisq.test() function from the base stats package (v4.4.0) to assess the associations between categorical variables. Effect size was evaluated using Cramér’s V via the CramérsV() function from the lsr library (v0.5.2). To evaluate trends in age-specific prevalence, we applied the chi-squared test for trends using the prop.trend.test() function from the stats package. All statistical tests were two-sided, and a p-value of < 0.05 was considered statistically significant. Given the number of exploratory comparisons, the results should be interpreted cautiously as no formal correction for multiple testing was applied; the primary focus remains on overall prevalence estimates.

## Results

### Population characteristics

Between January 1 and December 31, 2024, 3,695,039 individuals aged 5–99 years underwent eye examinations at Salud Digna diagnostic clinics across Mexico. Of this population, 1,529,784 individuals (41.4%) reported not using eyeglasses and had no prior record of an eye examination; this subset was used to identify cases of UREs. The mean age of this subpopulation was 39.3 years (SD = 19.5), with 921,062 (60.2%) females and 608,722 (39.8%) males.

Among these individuals, 1,520,575 (99.4%) reported that they had never undergone ophthalmologic evaluation. Additionally, 107,911 (7.0%) participants disclosed a prior diagnosis of diabetes, and 122,401 (8.0%) reported a history of high blood pressure. The most reported symptoms were blurred distant vision (909,000, 59.4%) and blurred near vision (750,118, 49%), whereas 324,364 (21.2%) individuals reported no visual symptoms. Table 1 summarizes the demographic and clinical characteristics of this subpopulation.

**Table 1:** Demographic characteristics of the outpatients included in this study

Carachteristic	*n*	%
Sex		
Female	2,382,045	62.6%
Male	1,425,502	37.4%
Age	42.16 ± 19.7	
< 10	108,986	2.9%
10–19	539,248	14.2%
20–29	566,093	14.9%
30–39	431,070	11.3%
40–49	623,186	16.4%
50–59	725,828	19.1%
60–69	512,225	13.4%
70–79	236,878	6.2%
≥ 80	64,033	1.7%
Previous eye examination		
Yes	1,887,759	49.6%
No	1,872,681	49.2%
Missed	47,107	1.2%
Previous opthalmologic revision		
Yes	56,730	1.5%
No	3,733,240	98.0%
Missed	17,577	0.5%
Use of glasses		
Yes	1,983,451	52.1%
No	1,776,989	46.7%
Missed	47,107	1.2%
Symptoms		
Blurred far vision	2,329,589	61.2%
Blurred near vision	1,879,567	49.4%
Headache	121,474	3.2%
Eye pain	76,123	2.0%
Itchy eyes	30,739	0.8%
None	679,827	17.9%
Diabetes		
Yes	366,087	9.6%
No	3,424,308	89.9%
Missed	17,152	0.5%
High blood pressure		
Yes	471,602	12.4%
No	3,315,287	87.1%
Missed	20,658	0.5%

### Uncorrected refractive errors

**Fig. 1:**
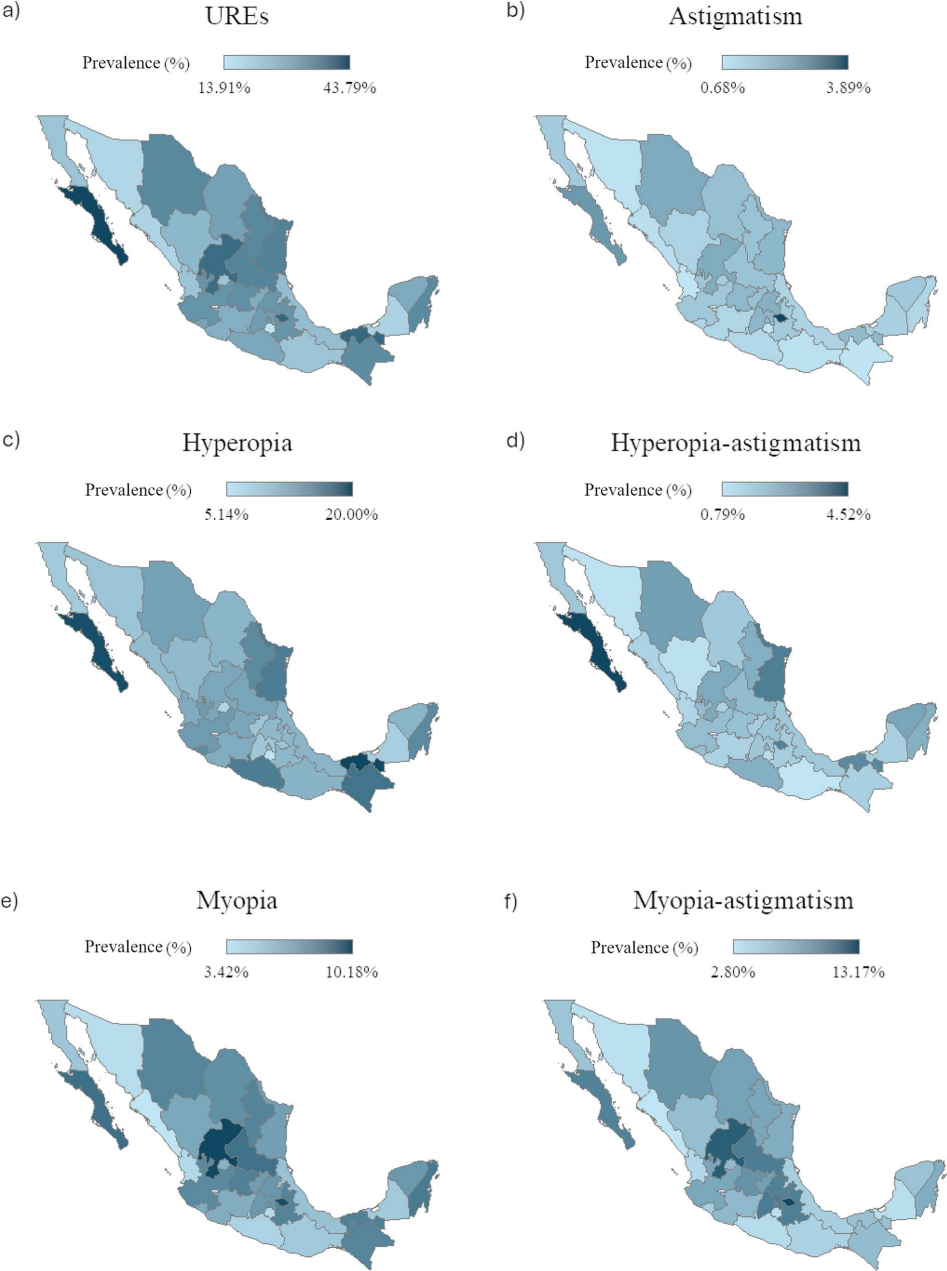
Clinic-based prevalence of uncorrected refractive errors (UREs) in Mexican outpatients. **a** Choropleth map of the clinic-based prevalence of URES in Mexican outpatients by state. **b** Choropleth map of the clinic-based prevalence of uncorrected astigmatism in Mexican outpatients by state. **c** Choropleth map of the clinic-based prevalence of uncorrected hyperopia in Mexican outpatients by state. **d** Choropleth map of the clinic-based prevalence of uncorrected hyperopia-astigmatism in Mexican outpatients by state. **e** Choropleth map of the clinic-based prevalence of uncorrected myopia in Mexican outpatients by state. **f** Choropleth map of the clinic-based prevalence of uncorrected myopia-astigmatism in Mexican outpatients by state. Abbreviations: URE= Uncorrected refractive error

We identified 980,718 URE cases, corresponding to a national clinic-based prevalence of 26.5% (95% CI: 26.5–26.59%). The highest prevalence was observed in Baja California Sur (43.8%, 95% CI: 42.99–44.59%), followed by Tlaxcala (38.1%, 95% CI: 37.19–38.98%) (Fig. 1a; Supplementary Table 1).

**Fig. 2:**
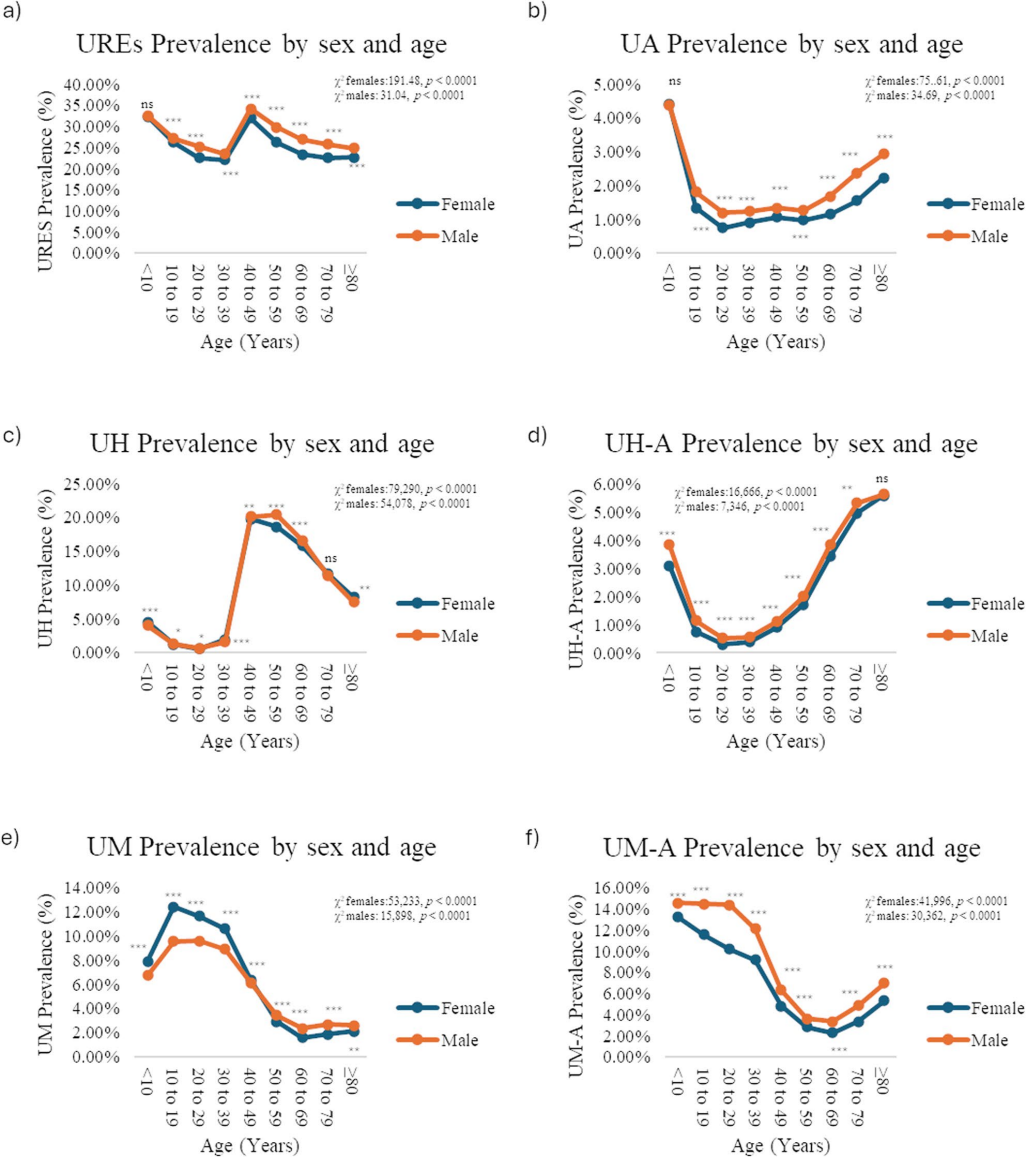
Clinic-based prevalence of uncorrected refractive errors (UREs) in Mexican outpatients by sex and age. **a** National clinic-based prevalence of URES in Mexican outpatients by sex and age. **b** National clinic-based prevalence of uncorrected astigmatism Mexican outpatients by sex and age. **c** National clinic-based prevalence of uncorrected hyperopia Mexican outpatients by sex and age. **d** National clinic-based prevalence of uncorrected hyperopia-astigmatism Mexican outpatients by sex and age. **e** National clinic-based prevalence of uncorrected myopia Mexican outpatients by sex and age. **f** National clinic-based prevalence of uncorrected myopia-astigmatism in Mexican outpatients by sex and age. Differences in age-specific clinic-based prevalence between sexes were evaluated using the chi-squared (χ2) test. Trends in age-specific clinic-based prevalence were evaluated using the chi-squared (χ2) test for trends. Asterisks indicate statistically significant differences: *p < 0.05, **p < 0.01, ***p < 0.001. Abbreviations: URE= Uncorrected refractive error, UA= Uncorrected astigmatism, UH= Uncorrected hyperopia, UH-A= Uncorrected hyperopia and astigmatism, UM= Uncorrected myopia. UM-A Uncorrected myopia and astigmatism

Stratification by sex and age showed a higher prevalence in males across all age groups (*p* < 0.0001), except < 10 years (*p* = 0.39). However, Cramér’s V indicated a weak association (V < 0.1). Among males, the prevalence peaked at 40–49 years (34.1%) and was lowest at 30–39 years (23.5%). Among females, the highest prevalence was observed in the < 10 years age group (32.3%) and the lowest in the 30–39 years age group (22.1%) (Fig. 2a; Supplementary Table 2).

### Uncorrected astigmatism

We identified 48,978 UA cases (national prevalence, 1.3%; 95% CI: 1.31–1.34%). Tlaxcala had the highest clinic-based prevalence (3.9%), followed by Baja California Sur (2.2%) (Fig. 1b; Supplementary Table 1).

Sex- and age-stratified analyses showed a higher clinic-based prevalence in males across all age groups (*p* < 0.0001), except for < 10 years (*p* = 0.8428), with a weak association (V < 0.1). In both sexes, the prevalence was highest in < 10 years (4.4%) and lowest in 20–29 years (males, 1.2%; females, 0.75%) (Fig. 2b; Supplementary Table 2).

### Uncorrected hyperopia

We identified 376,701 UH cases (10.2%, 95% CI: 10.16–10.23%) and 61,171 uncorrected hyperopia-astigmatism (UH-A) cases (1.66%, 95% CI: 1.64–1.67%). The highest UH clinic-based prevalence was in Tabasco (20%, 95% CI: 19.66–20.35%), whereas UH-A peaked in Baja California Sur (4.5%, 95% CI: 4.2–4.87%) (Figs. 1c–d; Supplementary Table 1).

Sex- and age-stratified analyses showed mixed patterns for UH: females had higher prevalence in < 10y (*p* < 0.0001), 30–39 (*p* < 0.0001), and ≥ 80 years (*p* = 0.007); males in 10–19 (*p* = 0.04), 20–29 (*p* = 0.03), 40–49 (*p* < 0.001), and 50–69 (*p* < 0.0001); and no difference in 70–79 years ((*p =* 0.05)). For UH-A, men had a higher prevalence across most age groups (10–69 *p* < 0.0001, 70–79 *p* < 0.001), except those aged ≥ 80 years (*p* = 0.84). All associations were weak (Cramér’s V < 0.1) (Figs. 2c–d; Supplementary Table 2).

Age-specific trends varied according to sex. For UH, prevalence peaked at 50–59 years in males (19.8%) and 40–49 years in females (19.3%), with the lowest prevalence in 20–29 years (< 1%). For UH-A, the highest prevalence occurred in those aged ≥ 80 years for both sexes (females: 5.39%; males: 5.63%), and the lowest in those aged 20–29 years (females: 0.28%; males: 0.5%) (Figs. 2c–d; Supplementary Table 2).

### Uncorrected myopia

We identified 235,285 μm cases (6.37%, 95% CI: 6.34–6.39%) and 258,583 uncorrected myopia-astigmatism (UM-A) cases (7.0%, 95% CI: 6.97–7.02%). The highest UM prevalence was in Zacatecas (10.2%), whereas UM-A peaked in Tlaxcala (13.2%) (Figs. 1e–f; Supplementary Table 1).

Sex- and age-stratified analyses showed higher UM prevalence in females < 50 years (*p* < 0.0001) and in males ≥ 50 years (50-79y *p* < 0.0001, ≥ 80 *p* < 0.001); UM-A was consistently higher in males across all age groups (*p* < 0.0001). The associations were weak (Cramér’s V < 0.1) (Figs. 2e–f; Supplementary Table 2).

Age-specific trends were similar for both conditions: UM peaked at 10–19 years (females: 11.97%; males: 9.2%) and was lowest at 60–69 years of age (females: 1.53%; males: 2.25%). UM-A was highest in the < 10 years age group (females: 12.8%; males: 14.1%) and lowest at 60–69 years (females: 2.21%; males: 3.2%) (Figs. 2e–f; Supplementary Table 2).

### Visual impairment

Using the WHO criteria, we identified 32,962 individuals with VI linked to UREs (national prevalence: 0.9%, 95% CI: 0.88–0.9%). The mean age was 49.3 years (SD = 25.9), with 60.1% of the participants being female. Most (98.4%) had never undergone ophthalmologic evaluation, and 15.7% reported diabetes and 15.7% reported hypertension. The most common symptoms included blurred distance vision (70.2%) and blurred near vision (56.7%), whereas 13.6% of patients reported no symptoms (Table 2).

**Table 2:** Demographic characteristics of outpatients included in this study who declared not using eyeglasses and had no prior record of an eye examination at the time of the eye examination

Carachteristic	*n*	%
Sex		
Female	1,068,719	60.1%
Male	708,270	39.9%
Age	39.5 ± 19.6	
< 10	82,409	4.6%
10–19	286,819	16.1%
20–29	264,147	14.9%
30–39	214,108	12.0%
40–49	337,177	19.0%
50–59	291,342	16.4%
60–69	184,449	10.4%
70–79	89,077	5.0%
≥ 80	27,461	1.5%
Previous eye examination		
Yes	72,105	4.1%
No	1,704,884	95.9%
Previous opthalmologic revision		
Yes	12,482	0.7%
No	1,764,228	99.3%
Missed	279	0.0%
Symptoms		
Blurred far vision	983,354	55.3%
Blurred near vision	818,431	46.1%
Headache	65,733	3.7%
Eye pain	31,805	1.8%
Itchy eyes	15,648	0.9%
None	394,964	22.2%
Diabetes		
Yes	129,451	7.3%
No	1,647,534	92.7%
Missed	4	0.0%
High blood pressure		
Yes	144,921	8.2%
No	1,630,117	91.7%
Missed	1,951	0.1%

**Fig. 3:**
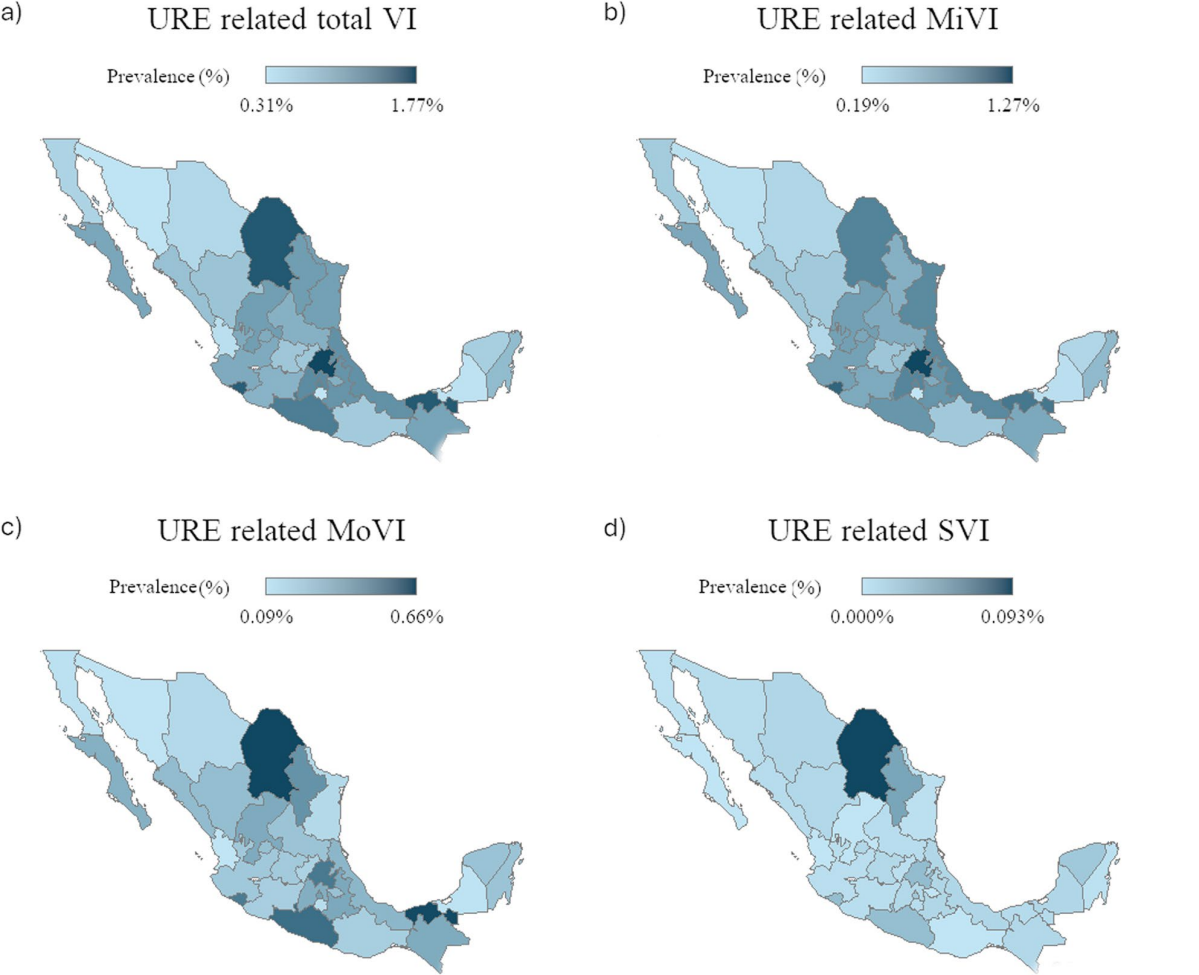
Clinic-based prevalence of URE-related visual impairment in Mexican outpatients. **a** Choropleth map of the clinic-based prevalence of URE-related visual impairment in Mexican outpatients by state. **b** Choropleth map of the clinic-based prevalence of URE-related mild visual impairment (MiVI) in Mexican outpatients by state. **c** Choropleth map of the clinic-based prevalence of URE-related moderate visual impairment (MoVI) in Mexican outpatients by state. **d** Choropleth map of the clinic-based prevalence of URE-related severe visual impairment (SVI) in Mexican outpatients by state. Abbreviations: URE= Uncorrected refractive error, VI= Visual impairment, MiVI= Mild visual impairment, MoVI= Moderate visual impairment, SVI= Severe visual impairment

The clinic-based prevalence was highest in Hidalgo (1.8%) and Coahuila (1.6%) (Fig. 3a; Supplementary Table 3). Sex- and age-stratified analysis showed that females had a higher prevalence in < 10y (*p* = 0.003), while males predominated in several groups (10–79y; 10-19y *p* = 0.0013, 20-29y *p* < 0.0001, 30-39y *p* < 0.001, 60-69y *p* = 0.01, 70-79y *p* = 0.042), although the associations were weak (Cramér’s V < 0.1). Prevalence peaked at ≥ 80 years (females: 6.1%; males: 6.2%) and was lowest at 40–49 years (females: 0.42%; males: 0.4%) (Fig. 3a; Supplementary Table 3).

**Fig. 4:**
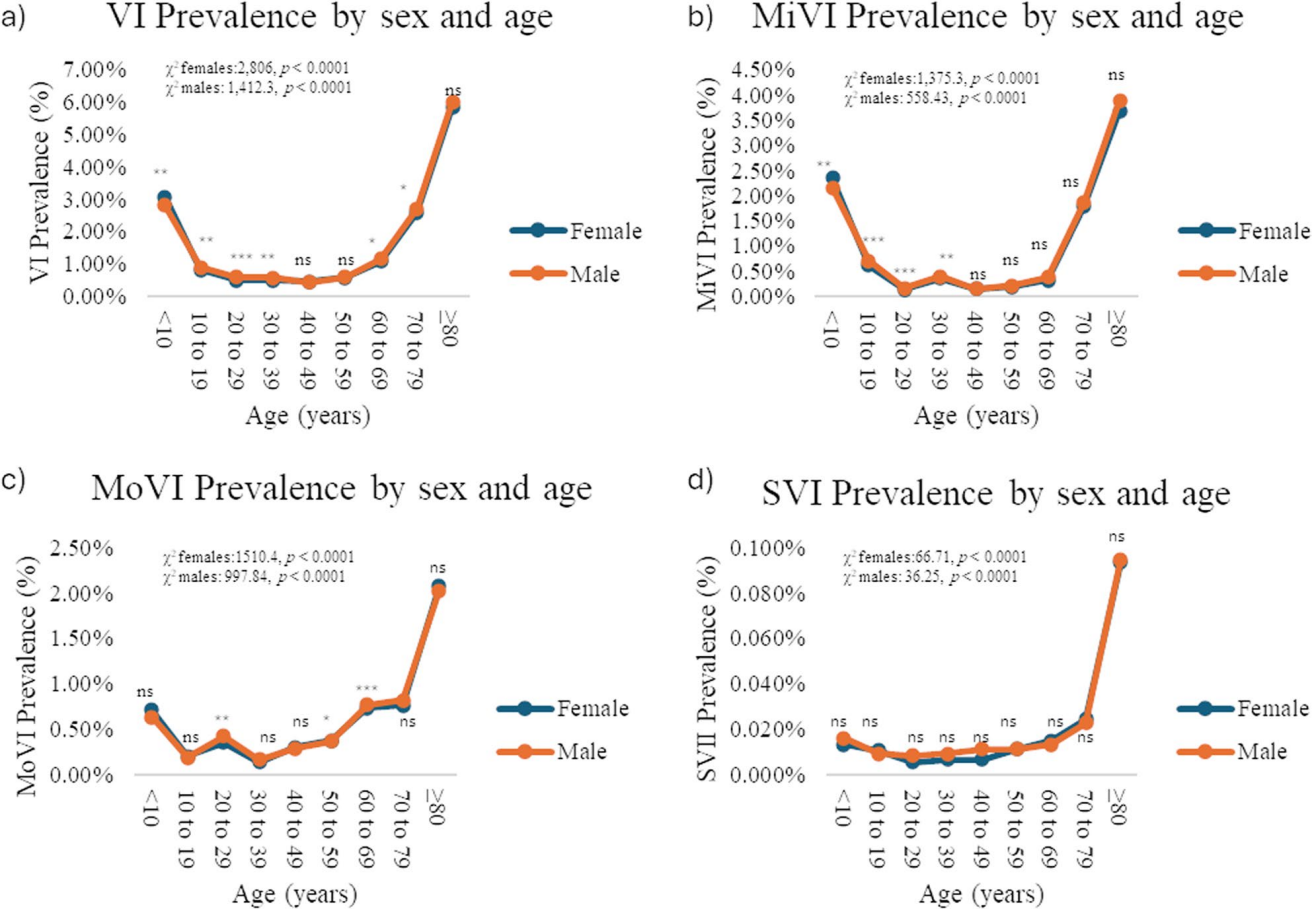
Clinic-based prevalence of URE-related visual impairment in Mexican outpatients by sex and age. **a** National clinic-based prevalence of URE-related visual impairment in Mexican outpatients by sex and age. **b** National clinic-based prevalence of URE-related mild visual impairment in Mexican outpatients by sex and age. **c** National clinic-based prevalence of URE-related moderate visual impairment in Mexican outpatients by sex and age. **d** National clinic-based prevalence of URE-related severe visual impairment in Mexican outpatients by sex and age. Differences in age-specific clinic-based prevalence between sexes were evaluated using the chi-squared (χ2) test. Trends in age-specific clinic-based prevalence were evaluated using the chi-squared (χ2) test for trends. Asterisks indicate statistically significant differences: *p < 0.05, **p < 0.01, ***p < 0.001. Abbreviations: VI= Visual impairment, MiVI= Mild visual impairment, MoVI= Moderate visual impairment, SVI= Severe visual impairment

Severity analysis identified 22,858 mild VI (MiVI), 9,683 moderate VI (MoVI), and 421 severe VI (SVI) cases, corresponding to clinic based prevalences of 0.62%, 0.26%, and 0.01%, respectively (Figs. 3b–d; Supplementary Table 3). Hidalgo had the highest MiVI prevalence (1.27%), whereas Coahuila led in MoVI (0.66%) and SVI (0.09%). Across sexes, ≥ 80 years showed the highest prevalence for all severity categories (MiVI, females 3.84%,; males: 4.06%), (MoVI, females: 2.15%; males: 2.12%,) (SVI, females: 0.09%; males 0.09%) (Fig. 4; Supplementary Table 4).

**Fig. 5:**
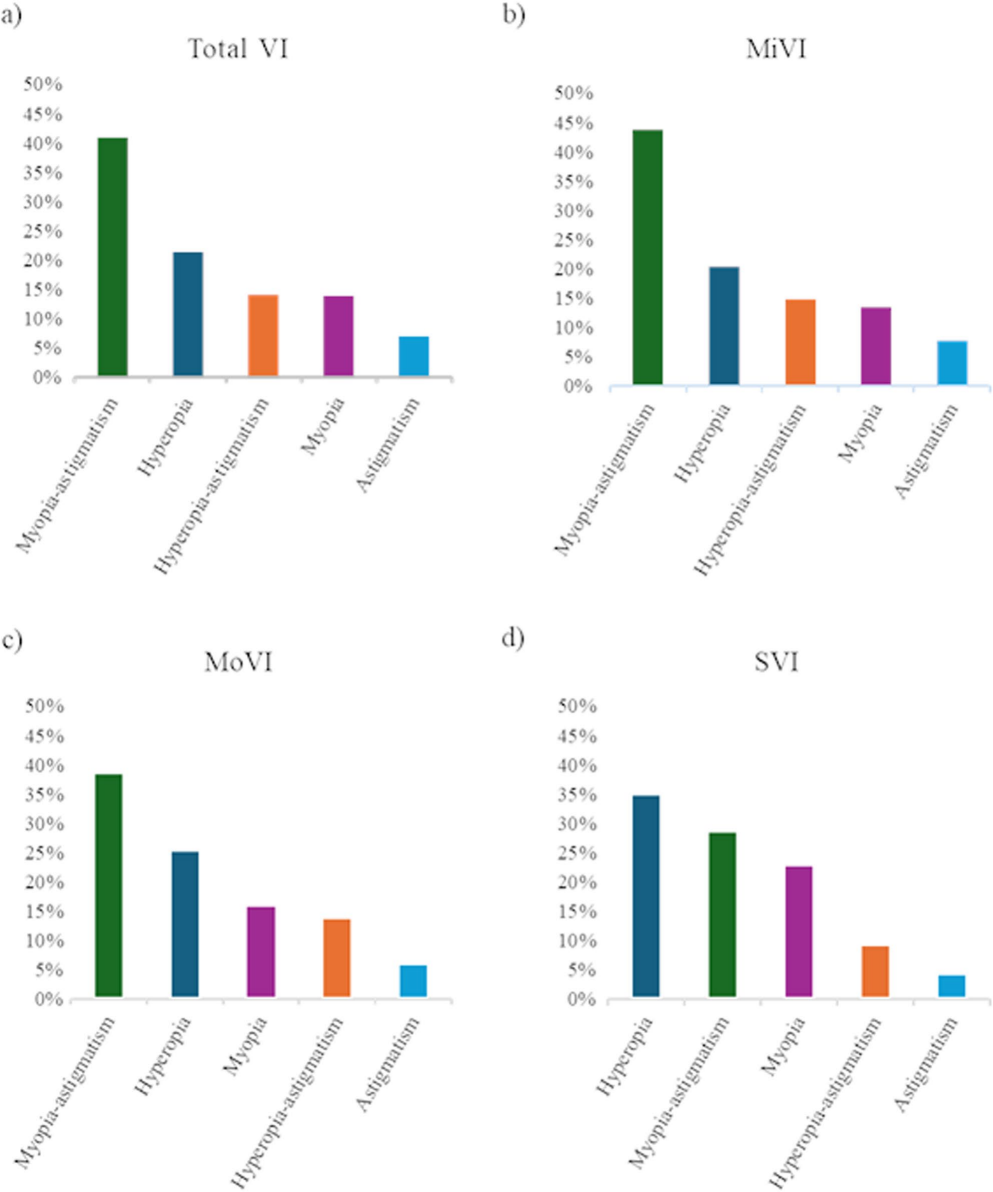
Visual impairment and associated URE in Mexican outpatients. **a** Global visual impairment and associated URE in Mexican outpatients. **b** Global mild visual impairment and its associated URE in Mexican outpatients. **c** Global moderate visual impairment and its associated URE in Mexican outpatients. **d** Global severe visual impairment and its associated URE in Mexican outpatients. Abbreviations: VI= Visual impairment, MiVI= Mild visual impairment, MoVI= Moderate visual impairment, SVI= Severe visual impairment

By refractive error type, myopia-astigmatism accounted for 43.7% of MiVI and 38.5% of MoVI cases, whereas hyperopia was the leading cause of SVI (35.4%) (Fig. 5; Supplementary Table 5).

## Discussion

The etiology of refractive errors remains incompletely understood, with both genetic and environmental factors being implicated. However, UREs are primarily a sociocultural phenomenon rather than a purely physiological one, reflecting systemic gaps in public health infrastructure and exerting substantial socioeconomic consequences. If left untreated, UREs may progress to SVI and, in some cases, blindness [10].

Multiple social determinants contribute to the burden of UREs, including limited health education, the absence of a preventive care culture, and restricted access to visual health services, particularly in low-income communities [10]. As Ono has observed, “blindness is not always prioritized by health and public health practices, especially when mortality indicators are used” [18]. These insights underscore the need for integrated, equity-focused approaches to eye care within national health systems.

In Mexico, few studies have comprehensively addressed the burden of UREs. This study included a large patient sample and broad geographic coverage, encompassing all 32 states of the country. Our objective was to characterize the current epidemiological profile of URE incidence and associated VI, thereby contributing to a more nuanced understanding of these conditions. This is particularly relevant in the post-pandemic context, where the impact of prolonged confinement has extended to visual health [19–21].

Importantly, demographic shifts, including population growth, aging, urbanization, and lifestyle changes, are expected to significantly increase the number of individuals living with ocular conditions, VI, and blindness in the coming decades. These trends pose substantial challenges for public health systems and underscore the urgency of implementing scalable, equitable eye care strategies [10].

### UREs incidence and VI

The substantial proportion of individuals who had never undergone an eye examination or ophthalmologic evaluation highlights critical gaps in preventive health culture, self-care education and access to visual health services. Additionally, the high prevalence of diabetes and hypertension, both major risk factors for ocular conditions [22], underscores the need to raise awareness and empower communities regarding eye care.

Our analysis revealed that UH was the most clinic-based prevalent URE. However, distinct age-related patterns have emerged. Notably, we observed a high clinic-based prevalence of UM and UM-A among children, consistent with the findings of Cao et al., [23] who identified myopia and astigmatism in young children as the leading global causes of URE. These conditions are also recognized as key risk factors for amblyopia, a visual disorder with lifelong consequences [24].

Our findings align with previous reports indicating that UREs are a significant concern among Mexican schoolchildren [25, 26]. This underscores the urgent need to establish and strengthen school-based screening programs for refractive errors, along with targeted training and educational initiatives for teachers and school health workers to address this issue.

Our findings revealed a persistently high clinic-based prevalence of UM and myopia with astigmatism (UM-A) up to 40 years of age, significantly affecting the most economically active segment of the population. This is particularly relevant given the WHO’s observation that “individuals and families are frequently pushed into a cycle of deepening poverty because of their inability to see well” [27].

In contrast, UH was most clinic-based prevalent among older adults, a population for whom VI is strongly associated with reduced quality of life, increased risk of cognitive decline, and heightened vulnerability to falls and fractures [28–30].

The regional disparities in the geographical distribution of UREs observed in this study may reflect differences in socioeconomic conditions, healthcare infrastructure, urbanization, and cultural attitudes toward preventive eye care. Further research is needed to quantify the impacts of these determinants.

States with higher incidence, such as Baja California Sur and Tlaxcala, may have limited access to optometry services or lower health literacy, which can delay corrective measures. Previous studies have shown that rural and low-income communities face significant barriers to eye care, including cost, availability of trained professionals, and awareness of visual health needs [10]. These findings underscore the importance of targeted strategies to reduce inequities and improve access to eye care in these communities.

Regarding VI, an 11.7% prevalence has been reported in an American cohort of adults aged ≥ 40 years [29]. In low-income communities in the United States, URE-related VI has been estimated at 8.2% [31]. In Latin America, approximately 7.2 million individuals live with URE-related VI, representing a regional prevalence of 1.4% [16], which is comparable to the national URE-related VI clinic-based prevalence observed in our study (0.9%). Our findings are consistent with those of Resnikoff et al., [32] who reported a disproportionate burden of URE-related VI in older populations.

Notably, our data show elevated clinic-based prevalence rates of URE-related VI in states such as Hidalgo and Coahuila, suggesting that individuals in these regions may not receive adequate eye care. Further investigation is required to identify the underlying factors contributing to these differences.

Unfortunately, direct comparisons between our findings and those of other national or international studies are limited because of differences in definitions and methodologies and the challenges of extrapolating from population-specific data.

### Strengths and limitations

A key strength of this study lies in its large, nationally representative sample encompassing all 32 Mexican states, which enhances the generalizability and relevance of the findings to the broader population. Additionally, standardized eye examination protocols were consistently applied across all clinics and were conducted exclusively by certified optometrists, ensuring methodological rigor.

In terms of VI classification, we utilized the WHO thresholds related to BCVA for descriptive purposes. Even though we omitted documented ocular comorbidities that could lead to VI, the possibility of residual misclassification cannot be entirely dismissed because of the lack of UCVA data in our dataset. Furthermore, presbyopia data, a significant refractive error particularly relevant to older adults [2], were missing and thus, not specifically examined in this study.

It is also crucial to acknowledge that the study population comprised outpatients who voluntarily sought diagnostic services at Salud Digna, representing a self-selected cohort that included both insured and uninsured individuals from diverse socioeconomic backgrounds. Thus, this cohort may not fully reflect socioeconomic gradients, urban versus rural residence, access to healthcare, and social security coverage of the Mexican population. Moreover, the lack of socioeconomic variables in our dataset limits the analysis of social gradients in UREs and VI clinic-based prevalence.

Although self-selection introduces potential bias, the large sample size facilitates robust stratified analyses and mitigates some of the limitations inherent in observational data. Consequently, findings derived from this dataset should be interpreted with caution within the context of this population, recognizing the potential influence of selection bias, as our results are not directly generalizable to the entire population of Mexico.

## Conclusions

Over the past three decades, the global prevalence of vision impairment due to UREs has declined; however, demographic shifts, particularly population growth and aging, have led to an increase in the absolute number of affected individuals.

To address the societal burden of VI, the WHO, through its *World Report on Vision*, has advocated for the integration of people-centered eye care into national health systems, with a strong emphasis on primary care [10]. This approach encompasses health promotion, prevention, treatment, and rehabilitation strategies.

In Mexico, there is a scarcity of data on refractive errors, particularly uncorrected ones, and the health information required for effective planning is inadequate.

In this study, our comprehensive nationwide analysis identified that 26.5% of outpatients seeking visual care in Salud Digna experienced UREs, with 0.9% suffering from associated VI. Hyperopia emerged as the most clinic-based prevalent URE, whereas myopia and myopia-astigmatism were notably common among children and young adults. Our research provides a crucial examination of the current epidemiological status of UREs in Mexico, intending to inform collaborative initiatives across sectors, both nationally and in culturally analogous regions.

Enhancing cooperation between the public and private sectors is crucial to empower individuals and communities regarding eye care needs and to formulate strategies that enhance access to visual health services. Additionally, this study aims to highlight the significance of addressing refractive errors due to their high prevalence and to alert both private organizations and the healthcare sector to the pressing need to provide adequate visual care and rehabilitation to those affected.

## Supplementary Information

Supplementary Material 1.

## Data Availability

The data supporting the findings of this study are available from the corresponding author upon reasonable requests. Owing to legal and ethical restrictions under the Mexican Federal Law on the Protection of Personal Data Held by Private Parties, access to the data is limited to ensure the privacy and confidentiality of the study participants. Requests will be evaluated according to the institutional policies and applicable regulations.

## Abbreviations

URE: Uncorrected refractive error
VI: Visual impairment
UM: Uncorrected myopia
UA: Uncorrected astigmatism
UH: Uncorrected hyperopia
UM-A: Uncorrected myopia with astigmatism
UH-A: Uncorrected hyperopia with astigmatism
MiVI: Mild visual impairment
MoVI: Moderate visual impairment
SVI: Severe visual impairment
